# Reply to: Death by community-based methicillin-resistant *Staphylococcus aureus*: case report

**DOI:** 10.62675/2965-2774.20240155-en

**Published:** 2024-10-31

**Authors:** Julia Lima Vieira, Tais Sica da Rocha, Francisco Bruno, Ruy Pezzi de Alencastro, Jefferson Pedro Piva

**Affiliations:** 1 Hospital Moinhos de Vento Porto Alegre RS Brazil Hospital Moinhos de Vento - Porto Alegre (RS), Brazil.; 2 Universidade Federal do Rio Grande do Sul Hospital das Clínicas de Porto Alegre Porto Alegre RS Brazil Hospital das Clínicas de Porto Alegre, Universidade Federal do Rio Grande do Sul - Porto Alegre (RS), Brazil.

To the Editor

We thank Scorza et al.^(1)^ for their thorough review of our report, as well as for the questions presented, which contributed greatly to the discussion and better understanding of the case. Now, we attempt to explain our diagnosis and answer the following questions:

## 1. PERFORMANCE OF HEAD COMPUTED TOMOGRAPHY OWING TO THE NEUROLOGICAL SIGNS OF THE PATIENT

We agree that an imaging test for diagnostic clarification was mandatory, so much so that it was requested immediately after the event of anisocoria. However, owing to the severity and instability of the patient, whose condition progressed to death within a few hours after hospitalization, it was not possible to transport the patient for the examination. Despite the impossibility of performing the examination, the autopsy performed elucidated the neurological event. The following is a summary of the autopsy findings regarding the central nervous system evaluation:

- Macroscopic evaluation: no changes. Brain weight = 1,374g.
- Microscopic evaluation of the brain and cerebellar parenchyma: edema and mild ischemic degenerative changes.
- Microscopic evaluation of the meninges: slight edema and congestion.

Therefore, according to these findings, the brain changes were ischemic and secondary to refractory shock. No signs of hemorrhage, hematoma, venous thrombosis, meningitis or encephalitis were identified.

Unfortunately, owing to the limitations imposed for case reports, we were not able to present all the relevant and clarifying data from the autopsy. Thanks to your questions, we have the opportunity to provide a more detailed description of the autopsy findings of greatest interest in a complementary manner.

- Microscopic findings of the right and left lungs: the lung parenchyma was massively compromised by coagulation necrosis associated with massive hemorrhage and numerous bacterial colonies that had profusely invaded the tissues. […] The bronchial tree showed extensive multifocal transmural coagulation necrosis associated with numerous bacterial colonies […]. The pleura showed diffuse coagulation necrosis associated with numerous bacterial colonies. The presence of septic bacterial emboli in small vessels was noted. In addition, edema and hemorrhage with bacterial colonies and intra-alveolar fibrin deposition in the few apical residual nonnecrotic lung areas were observed.
- Microscopic findings of the pharynx, larynx and trachea: there were extensive areas of ulcerated necrosis of the mucosa associated with numerous bacterial colonies. […] Edema and congestion were also observed.
- Mediastinal lymph nodes: foci of coagulation necrosis in the lymph node medullary zone were present. Small- and medium-sized vessels in the lymph node hilum and adjacent adipose tissue contained septic emboli with bacterial colonies.

## 2. POSSIBILITY OF COMMUNITY-ACQUIRED METHICILLIN--RESISTANT *STAPHYLOCOCCUS AUREUS* (GRAM-POSITIVE COCCUS) AS A CAUSE OF TISSUE NECROSIS

At autopsy, numerous bacterial colonies were observed in the upper and lower respiratory tract, and the significant predominance of community-acquired methicillin-resistant *Staphylococcus aureus* (CA-MRSA [gram-positive coccus]) was the cause of tissue necrosis in relation to *Haemophilus influenzae* (gram-negative bacillus) infection. Unfortunately, the bacteriological characteristics of CA-MRSA, such as the presence of Panton-Valentine leukocidin gene variants, could not be evaluated owing to the unavailability of a specific polymerase chain reaction test.

## 3. POSSIBLE INVOLVEMENT OF SEVERE ACUTE RESPIRATORY SYNDROME CORONAVIRUS 2 AS AN ETIOLOGICAL AGENT

As mentioned, no test was performed for severe acute respiratory syndrome coronavirus 2 (SARS-CoV-2); however, there was a record that the patient had received, more than 30 days prior, two doses of the SARS-CoV-2-specific vaccine according to the vaccination schedule of the Unified Health System (SUS - *Sistema Único de Saúde*). Although the possibility of co-infection with SARS-CoV-2 exists, the incidence of severe forms of coronavirus disease 2019 (COVID-19) have been significantly reduced.^(2)^ In addition, the autopsy report highlighted the extensive presence of bacterial colonies and diffuse septic emboli in the tissues of the upper and lower respiratory tracts, causing hemorrhage, necrosis and air leakage, confirming the direct involvement of bacteria as an etiological agent in pulmonary necrosis.

## 4. POSSIBILITY OF PREVIOUS COAGULOPATHY

The patient had been described as being healthy, with no previous hospitalizations and no history of previous persistent or abnormal bleeding. Disseminated intravascular coagulation is a severe, acquired condition characterized by systemic activation of the hemostatic system, resulting in excessive deposition of thrombin and leading to microvascular thrombi. In addition, platelet consumption and coagulation factor depletion promote an imbalance between the fibrinolytic and antifibrinolytic systems and are associated with severe bleeding.^(3)^ Because the patient had alterations in all stages of coagulation, identified both by laboratory tests and on thromboelastograms (severe deficiency of intrinsic and extrinsic pathway factors, fibrinogen deficiency, and platelet deficiency/dysfunction), in the presence of a compatible triggering factor (refractory septic shock), the diagnosis of disseminated intravascular coagulation associated with consumption coagulopathy secondary to massive hemorrhage is reasonable.

We thank you, once again, for the questions, which allowed us to provide a more detailed description of the case and enriched the discussion.
