# Supplementary material for: Reply to: Death by community-based methicillin-resistant Staphylococcus aureus: case report
Source: Crit Care Sci. 2024 Oct 31;36:e20240155en. doi: 10.62675/2965-2774.20240155-en (PMC11634237; doi:10.62675/2965-2774.20240155-en)
Supplement: Translation

# Translation: Resposta para: Morte por *Staphylococcus aureus* comunitário resistente à meticilina: relato de caso

**DOI:** 10.62675/2965-2774.20240155-pt

**Authors:** Julia Lima Vieira, Tais Sica da Rocha, Francisco Bruno, Ruy Pezzi de Alencastro, Jefferson Pedro Piva

**Affiliations:** 1 Porto Alegre RS Brasil Hospital Moinhos de Vento - Porto Alegre (RS), Brasil.; 2 Porto Alegre RS Brasil Hospital das Clínicas de Porto Alegre, Universidade Federal do Rio Grande do Sul - Porto Alegre (RS), Brasil.

Ao Editor

Agradecemos a Scorza et al.^(1)^ pela minuciosa revisão de nosso relato, assim como pelos questionamentos apresentados, que contribuem muito para a discussão e o melhor entendimento do caso. A seguir, tentamos justificar e responder os questionamentos apresentados:

## 1. REALIZAÇÃO DE TOMOGRAFIA COMPUTADORIZADA DE CRÂNIO EM RAZÃO DOS SINAIS NEUROLÓGICOS DO PACIENTE

Evidentemente concordamos que um exame de imagem para elucidação diagnóstica era mandatório, tanto que foi solicitada imediatamente após o evento da anisocoria. Entretanto, devido à gravidade e à instabilidade do paciente, que evoluiu ao óbito em poucas horas após a internação hospitalar, não foi possível realizar o transporte para a realização do exame. Apesar da impossibilidade da realização do exame, a necropsia realizada foi elucidativa quanto ao evento neurológico. Transcrevemos a descrição do laudo de necropsia quanto à avaliação do sistema nervoso central:

- Avaliação macroscópica: sem alterações. Cérebro pesando 1.374g.
- Avaliação microscópica do parênquima cerebral e cerebelar: edema e alterações degenerativas isquêmicas discretas.
- Avaliação microscópica das meninges: discretos edema e congestão.

Portanto, conforme a descrição anatomopatológica, as alterações cerebrais foram isquêmicas e secundárias ao choque refratário. Não foram identificados sinais de hemorragia, hematoma, trombose venosa, meningite ou encefalite.

Infelizmente, em função da limitação imposta para relatos de casos, não pudemos apresentar todos os dados relevantes e esclarecedores da necropsia. Graças aos seus questionamentos, temos oportunidade de apresentar de forma complementar a descrição mais detalhada das partes de maior interesse do laudo da necropsia:

- Achados microscópicos do pulmão direito e esquerdo: parênquima pulmonar maciçamente comprometido por necrose de coagulação associada à volumosa hemorragia e numerosas colônias bacterianas que invadem profusamente os tecidos. […] Árvore brônquica apresentando necrose de coagulação transmural extensa multifocal, associada a numerosas colônias bacterianas […]. Pleura com necrose de coagulação difusa, associada a numerosas colônias bacterianas. Presença de êmbolos sépticos bacterianos em vasos de pequeno calibre. Edema e hemorragia com colônias bacterianas de deposição intra-alveolar de fibrina nas escassas áreas pulmonares não necróticas residuais apicais.
- Achados microscópicos da faringe, laringe e traqueia: extensas áreas de necrose ulcerada da mucosa, associada a numerosas colônias bacterianas. […] Edema e congestão.
- Linfonodos mediastinais: presença de focos de necrose de coagulação em zona medular linfonodal. Êmbolos sépticos com colônias bacterianas em vasos de pequeno e médio calibre em hilo linfonodal e adiposo adjacente.

## 2. *STAPHYLOCOCCUS AUREUS* RESISTENTE À METICILINA DA COMUNIDADE (COCO *GRAM*-POSITIVO) COMO CAUSADOR DA NECROSE DOS TECIDOS

Na necrópsia, observaram-se numerosas colônias bacterianas no trato respiratório superior e inferior, sendo facilmente identificável, por meio de características morfológicas e de coloração, o predomínio significativo de *Staphylococcus aureus* resistente à meticilina da comunidade (CA-MRSA [coco *Gram*-positivo]) como causador da necrose dos tecidos em relação a *Haemophilus influenzae* (bacilo *Gram*-negativo). Infelizmente, características bacteriológicas de CA-MRSA, como a identificação da variante carreadora dos genes de leucocidina de Panton-Valentine, não puderam ser avaliadas, devido à indisponibilidade de exame de reação em cadeia da polimerase específico.

## 3. POSSÍVEL ENVOLVIMENTO DO CORONAVÍRUS DA SÍNDROME RESPIRATÓRIA AGUDA GRAVE 2 COMO AGENTE ETIOLÓGICO

Como referido, não foi realizado teste para coronavírus da síndrome respiratória aguda grave 2 (SARS-CoV-2), porém, havia registro de que o paciente recebera, há mais de 30 dias, duas doses da vacina específica, conforme calendário vacinal do Sistema Único de Saúde (SUS). Apesar de não se excluir a possibilidade de coinfecção pelo vírus da doença pelo coronavírus 2019 (COVID-19), reduzem-se de forma significativa as formas graves da doença.^(2)^ Além disso, a descrição do exame anatomopatológico enfatiza a volumosa presença de colônias bacterianas e êmbolos sépticos difusamente em tecidos do trato respiratório superior e inferior, provocando hemorragia, necrose e extravasamento aéreo, confirmando o envolvimento direto da etiologia bacteriana na necrose pulmonar.

## 4. POSSIBILIDADE DE COAGULOPATIA PRÉVIA

O paciente tinha descrição de ser previamente hígido, sem internações prévias, sem história de sangramento prévio persistente ou anormal. A coagulação intravascular disseminada é uma condição severa, adquirida, caracterizada pela ativação sistêmica do sistema hemostático resultando em deposição excessiva de trombina e levando a trombos microvasculares. Além disso, o consumo de plaquetas e a depleção de fatores de coagulação promovem desbalanço entre sistemas fibrinolíticos e antifibrinolíticos, estando associados a sangramento grave.^(3)^ Visto que o paciente apresentava alteração de todas etapas da coagulação, identificada tanto por conta dos exames laboratoriais quanto no tromboelastograma (deficiência acentuada de fatores da via intrínseca e extrínseca; deficiência de fibrinogênio; deficiência/disfunção plaquetária), em vigência de um fator gatilho compatível (choque séptico refratário), o diagnóstico de coagulação intravascular disseminada associada à coagulopatia de consumo secundária à hemorragia maciça pode ser realizado com segurança.

Agradecemos, uma vez mais, aos questionamentos, os quais permitiram uma descrição mais detalhada do caso e o enriquecimento da discussão.
